# Understanding and breaking the intergenerational cycle of abuse in families enrolled in routine mental health services: study protocol for a randomized controlled trial and two non-interventional trials investigating mechanisms of change within the UBICA II consortium

**DOI:** 10.1186/s13063-021-05653-3

**Published:** 2021-10-28

**Authors:** C. Neukel, F. Bermpohl, M. Kaess, S. Taubner, K. Boedeker, K. Williams, A. Dempfle, S. C. Herpertz, the UBICA-II consortium, Sabine C. Herpertz, Corinne Neukel, Felix Bermpohl, Michael Kaess, Romulad Brunner, Svenja Taubner, Jana Volkert, Anna Georg, Leonie Fleck, Anna Fuchs, Tabea von der Lühe, Emilia Mielke, Peter Parzer, Franz Resch, Corinna Roth, Fabian Seeger, Marc Wenigmann, Katharina Williams, Christian Banzhaf, Katja Boedeker, Eva Brandl, Katja Dittrich, Catherine Hindi-Attar, Dorothea Kluczniok, Irene Sophia Plank, Catherina Reuter, Judith Ratayczak, Nikola Schoofs, Sybille Winter, Katja Bertsch

**Affiliations:** 1 Department of General Psychiatry, Center for Psychosocial Medicine, Heidelberg University, Heidelberg, Germany; 2 Department of Psychiatry and Psychotherapy, Berlin Institute of Health, Charité Universitätsmedizin Berlin, Corporate Member of Freie Universität Berlin, Humboldt-Universität Zu Berlin, Campus Charité Mitte, Berlin, Germany, Berlin, Germany; 3 Department of Child and Adolescent Psychiatry, Center for Psychosocial Medicine, Heidelberg University, Heidelberg, Germany; 4 University Hospital of Child and Adolescent Psychiatry and Psychotherapy, University of Bern, Bern, Switzerland; 5 Department of Psychosocial Prevention, University Hospital of Heidelberg, Heidelberg, Germany; 6 Charité – Universitätsmedizin Berlin, corporate member of Freie Universität Berlin, Humboldt-Universität zu Berlin, and Berlin Institute of Health (BIH), Campus Virchow-Klinikum, Department of Child and Adolescent Psychiatry, Psychosomatics and Psychotherapy, Berlin, Germany; 7 Institute of Medical Informatics and Statistics, University Hospital Schleswig-Holstein, Campus Kiel, Kiel, Germany

**Keywords:** Parenting, Mentalization-based therapy, Early life maltreatment, Prevention, Parent-child, Social cognition, Dyadic synchrony, Cluster randomized controlled trial

## Abstract

**Background:**

Parents’ mental illness (MI) and parental history of early life maltreatment (ELM) are known to be significant risk factors for poor parenting while poor parenting is a crucial mediator of the intergenerational continuity of child maltreatment. Hence, maltreatment prevention programs for families with an MI parent, which pay particular attention to experiences of ELM in the parent, are urgently needed. Parental mentalizing was previously found to mediate successful parenting. Interventions aimed at improving the parental mentalizing capacity reduced maltreatment risk in parents. The aim of the present study is to investigate the effectiveness of a mentalization-based parenting-counseling in acutely mentally ill parents currently treated at a psychiatric hospital.

**Methods:**

Mentalization-based parenting-counseling (MB-PC) vs. enhanced standard clinical care (SCC+) will be administered in a cluster-randomized-controlled trial (RCT). Patients treated at psychiatric hospitals with children between 1.5 and 15 years will be included in the trial. MB-PC will be administered as a 12-h combined individual and group program enriched by social counseling (over a course of 5 weeks) as add-on to standard clinical care, while the control condition will be standard clinical care plus a 90-min psychoeducation workshop on positive parenting. Primary efficacy endpoint is self-reported parenting practices at follow-up. Embedded within the RCT will be two sub-studies investigating social cognition and dyadic synchrony as biobehavioral mechanisms of change.

**Discussion:**

The main goal of the present study is to investigate ways to break the intergenerational continuity of maltreatment by assessing the benefits of a prevention program which aims at improving parenting in vulnerable mothers and fathers. MB-PC is a short, low-cost intervention which can be delivered by nurses and social workers and is applicable to MI patients with children with a broad range of diagnoses. If it is shown to be effective, it can be directly implemented into standard psychiatric hospital care thereby providing help to prevent child maltreatment.

**Trial registration:**

German Clinical Trials Register DRKS00017398. Registered on 5 July 2019

## Administrative information

Note: the numbers in curly brackets in this protocol refer to SPIRIT checklist item numbers. The order of the items has been modified to group similar items (see http://www.equator-network.org/reporting-guidelines/spirit-2013-statement-defining-standard-protocol-items-for-clinical-trials/).

**Table:** 

Title {1}	Understanding and breaking the intergenerational cycle of abuse in families enrolled in routine mental health and welfare services: study protocol for a randomized controlled trial and non-interventional trials investigating mechanisms of change within the UBICA II consortium
Trial registration {2a and 2b}.	Registry: German Clinical Trials Register Trial Identifier: DRKS00017398
Protocol version {3}	Study Protocol Version 1.6, 26.04.2021
Funding {4}	This study is funded by the German Federal Ministry of Education and Research (Funding number: 01KR1803B, 01KR1803C). The Federal Ministry of Education and Research was neither involved in the design of the study nor in collection, analysis, and interpretation of data or in writing the manuscript.
Author details {5a}	CN: Department of General Psychiatry, Center for Psychosocial Medicine, Heidelberg University, Heidelberg, Germany. FB: Department of Psychiatry and Psychotherapy, Berlin Institute of Health, Charité Universitätsmedizin Berlin, Corporate Member of Freie Universität Berlin, Humboldt-Universität Zu Berlin, Campus Charité Mitte, Berlin, Germany, Berlin, Germany. MK: Department of Child and Adolescent Psychiatry, Center for Psychosocial Medicine, Heidelberg University, Heidelberg, Germany and University Hospital of Child and Adolescent Psychiatry and Psychotherapy, University of Bern, Bern, Switzerland. ST: Department of Psychosocial Prevention, University Hospital of Heidelberg, Heidelberg, Germany. KB: Charité – Universitätsmedizin Berlin, corporate member of Freie Universität Berlin, Humboldt-Universität zu Berlin, and Berlin Institute of Health (BIH), Campus Virchow-Klinikum, Department of Child and Adolescent Psychiatry, Psychosomatics and Psychotherapy, Berlin, Germany. AD: Institute of Medical Informatics and Statistics, University Hospital Schleswig-Holstein, Campus Kiel, Kiel, Germany. SCH: Department of General Psychiatry, Center for Psychosocial Medicine, Heidelberg University, Heidelberg, Germany.
Name and contact information for the trial sponsor {5b}	Heidelberg University Hospital, Im Neuenheimer Feld 325, 69120 Heidelberg, Germany
Role of sponsor {5c}	The sponsor and funders do not have any authority over research activities.

## Introduction

### Background and rationale {6a}

Poor parenting is a crucial mediator of the intergenerational continuity of child maltreatment. Parents’ mental illness (MI) and parental history of early life maltreatment (ELM) are known to be the most significant risk factors in this vicious cycle of abuse: MI and a history of ELM are both associated with child abuse potential [1]. Ten years ago a guidance of the World Psychiatric Association (WPA) has been dedicated to the protection and promotion of mental health in children of persons with severe mental disorders indicating that not the diagnosis but severity and chronicity of psychopathology confer an increased risk for child maltreatment [2]. Furthermore, it has been shown that ELM alone does not significantly impact on parenting behavior but that mothers with a history of MI and severe ELM show a particularly high risk of low maternal sensitivity in interaction with their children [3, 4]. About 3.8 million minors in Germany live in families with an MI parent [5]. In a nationwide survey of mental health professionals at psychiatric hospitals in Germany, 38.3% of the surveyed evaluated their patients as being impaired in parenting, at least sometimes, and 52.3% of the surveyed estimated the mental health of their patients’ children to be jeopardized [6]. Furthermore, MI parents often consider their children as having more psychological/behavioral problems and express a higher need for help in parenting than a healthy control group [5]. It is important to note that investigations of parenting report statistical associations in large samples. Many parents with severe mental illnesses show excellent caregiving that means a parental MI or experience of ELM do not necessarily lead to poor parenting and it is not inevitable that there is a negative impact on the children’s development. Nevertheless, children living in a family with an MI parent run a higher risk of being affected by various modes of ELM [7] and of developing psychopathology [8]. Although studies on parent-child-interaction and maltreatment of minors have been widely restricted to mothers, it has also been shown that ELM in fathers predicts harsh parenting [9] and parental neglect [10]. Additionally, MI such as depression in fathers is associated with, e.g., more withdrawn parental behavior [11] and increased risk of child maltreatment [12]. Hence, prevention programs for families with an MI parent, which pay particular attention to experiences of ELM in the parent, are urgently needed.

The Guidelines of the World Psychiatric Association (WPA) outline impaired responsiveness to the child’s needs, emotional unavailability, irritability, and disturbed behaviors as common pathways through which MI in parents, irrespective of the specific diagnosis, impact on their child’s well-being [2]. These pathways interact strongly with poor mentalizing, which has been shown to be a significant mediator of poor parenting and to be particularly impaired by ELM [13]. Thus, mentalization-based (MB) interventions foster social cognition in parents and provide an important and at the same time trainable basis on which to improve parents’ capacity to understand the meaning of their child’s behavior, resulting in more adaptive interactions and prevention of maltreatment [14].

A meta-analysis on the effects of parenting programs on child maltreatment prevention encompassing 37 studies provided evidence of positive effects and reduced risk of maltreatment [15]. Via own literature search, we found eleven different prevention programs that aim to enhance mentalizing in mothers with MI [16–21], in prison [22] or at other risk, such as teenage mothers [23], as well as in families [24, 25] and in caregivers of inpatient psychiatric children [26]. The efficacy of four of these approaches that aim to prevent abuse by improving mentalizing in parents has been demonstrated in six randomized-controlled trials (RCTs) [16, 21, 22, 25, 27, 28], and a further two RCTs are currently underway [26, 29]. Previous studies show that mentalization-based (MB) approaches can be delivered by nurses and social workers (e.g., [23]). A meta-analysis [30] including 10 studies encompassing 1628 mother-child dyads with 0–6-year-old children examined the effectiveness of interventions that aimed at promoting maternal sensitivity and reflective functioning. It found that interventions for high-risk families produced the most beneficial effects, with large effect sizes (*d* = 1.5). The authors pointed out that studies should include children in addition to infants and should extend measurements of child outcomes. In sum, data provide evidence for the efficacy of MB approaches in preventing parents from abusing their children, whether they are delivered for individuals, families, or in groups. Previous studies have focused on mothers or parent-couples, leaving open the question of whether they are also successful with fathers. Even more importantly, although several studies have been performed in inpatient treatment settings for addiction [18, 19], so far, no study has been conducted within general psychiatric hospitals for acutely MI parents with different diagnoses of the whole spectrum of mental disorders (e.g., depression, anxiety disorders, borderline personality disorder, addiction, schizophrenia).

We conceptualized a mentalization based-parenting counseling (MB-PC) to improve social cognition in parents in response to their child. In order to evaluate and optimize therapeutic interventions, it is particularly important to understand the biobehavioral mechanisms that underlie these processes and may, thus, induce, influence or moderate the effectiveness of this intervention. Two possible mechanisms of change regarding parenting are social cognition and dyadic synchrony:

First, both affective and cognitive components of social cognition are required for positive parenting: Affective components of social cognition concern the sharing of the child’s emotion (i.e., empathy) and the feelings of warmth and care (i.e., compassion). Cognitive components of social cognition, commonly conceptualized under the terms mentalizing, theory of mind (ToM), or cognitive perspective taking, comprise inferring and reasoning about the child’s cognitive and affective states (i.e., cognitive and affective ToM) [31]. Compromised social cognition in individuals with mental illness and ELM may be a key factor contributing to poor parenting, which impacts on child well-being and mediates the intergenerational continuity of child maltreatment [32]. To our knowledge, to date, no study has addressed the question of which components of social cognition may specifically mediate effects of mentalization-based parenting interventions.

Second, dyadic synchrony is commonly found in well-adjusted children and their parents and has been associated with favorable developmental outcomes [33, 34]. According to the biobehavioral model of parent-child synchrony [35], dyadic synchrony represents a dynamic regulatory process by which hormonal, physiological, and behavioral cues are exchanged between parent and child during social contact in order to jointly pull each other toward a baseline level characterized by greater stability in the system. In a synchronous relationship, when a child becomes distressed, the caregiver will be able to regulate his/her own feelings of discomfort and adopt a soothing demeanor, helping the child to regain emotional balance [36]. The parental ability to mentalize has been linked to observational measures of dyadic interactional quality such as higher sensitivity and lower hostility [37], and these links have also been found in the context of parental ELM and psychopathology [38, 39]. Biobehavioral synchrony could be affected by MB interventions but to date it has not been studied as a mechanism of change.

Taken together, research up to now shows that parental ELM and MI are associated with poor parenting while poor parenting is a risk factor for child maltreatment. MB interventions for parents have been identified as effective in reducing the maltreatment risk for the next generation while no study so far has addressed a group of acutely MI patients currently treated at general psychiatric hospitals. Furthermore, social cognition and dyadic synchrony are of importance for positive parenting and could be mechanisms of change in MB interventions. The present study therefore aims at investigating the effectiveness of an MB intervention on parenting quality in MI parents and possible biobehavioral mechanisms associated with it. The study is part of the multicenter consortium UBICA II (“Understanding and Breaking the Intergenerational Cycle of Abuse in Families Enrolled in Routine Mental Health and Welfare Services”) situated at three sites (Aachen, Berlin, Heidelberg) and subsuming several studies across Germany, led by the managing site at the Department of General Psychiatry at the University Hospital Heidelberg.

## Objectives {7}

The aim of this trial, which is embedded in the consortium UBICA II, is to investigate ways to break the intergenerational cycle of abuse by preventing abuse or neglect in children of MI parents or other parents at high risk of child maltreatment. To this end, the study will test real-life effectiveness of a short (12 h) mentalization-based parental counseling (MB-PC) program integrated into standard clinical treatment in two psychiatric hospitals in a randomized controlled trial (RCT). The clinical relevance of social cognition and parent-child synchrony as mediators of response to MB-PC will further be investigated in two sub-studies of the RCT.

The primary hypothesis of the RCT is that impaired parenting can be improved more by a 12-h combined individual and group MB-PC program enriched by social counseling as add-on to standard clinical care compared to enhanced standard clinical care (standard clinical care plus psychoeducation on positive parenting (SCC+)). Furthermore, we hypothesize that MB-PC-related behavioral and neural changes in social cognition as well as changes in biobehavioral synchrony will mediate the effects of the MB-PC intervention and that ELM will moderate the effects of MB-PC on social cognition and biobehavioral synchrony.

## Trial design {8}

Cluster-randomized, open-label (partially observer-blind), active-control, parallel group superiority clinical trial, with MB-PC groups of 4–8 patients as clusters and 1:1 allocation ratio for clusters.

## Methods: participants, interventions, and outcomes

### Study setting {9}

The Department of General Psychiatry at the University Hospital Heidelberg has 127 in-patient places, 58 day-clinic places, and 2000 cases per year to whom high-frequent out-patient treatment is offered providing mental health care for all citizens of a medium-sized high-socioeconomic status town. Patients come from the whole spectrum of mental disorders with the exception of detoxification treatment. The Psychiatric University Hospital Charité at St. Hedwig Hospital in Berlin has 156 in-patient places, 66 day-clinic places, and 9700 cases per year to whom high-frequent out-patient treatment is provided. It is situated in a metropolitan region with a rather low socioeconomic status, socially troubled areas, high rates of immigrants and addiction disorders. Notably, in-patient and day treatment is offered to acutely ill patients of moderate up to severe degree of illness in Germany with 94% in Heidelberg and 84% in Berlin having entered treatment deliberately. The mean length of stay in in-patient treatment is 25.6 days in Heidelberg and 18.8 days in Berlin, and 28.1 days in day treatment in Heidelberg and 23.2 days in Berlin.

### Eligibility criteria {10}

All patients with a child between 1.5 and 15 years who are in treatment at the Department of General Psychiatry at the University Hospital Heidelberg or the Psychiatric University Hospital Charité at St. Hedwig Hospital in Berlin and have regular contact to their child will be asked to participate in the study. Participation in a clinical trial requires capacity for informed consent and excludes those patients under compulsory treatment. Further exclusion criteria are those which are incompatible with psychotherapy, that is, IQ < 70, severe cognitive impairment of various etiology, and severe impairment in social competency that is not compatible with group interventions according to clinical judgment (i.e., aggressive behavior that endangers other participants or disruptive behavior making execution of the group impossible; patients are excluded from participation in the study if they are not allowed to take part in other group therapies taking place at the in- and outpatient units due to aggressive or disruptive behaviors). The sub-studies comprise additional inclusion criteria due to hormonal analysis and functional magnetic resonance (fMRI) measurements. Informed assent of children (from the age of 6) is necessary for the assessment of children (self-report questionnaires as outcome measures) and their participation in sub-studies. However, participation of a patient in the main trial without assessment of his/her child is also possible.

### Who will take informed consent? {26a}

Informed consent will be obtained by psychologists and medical doctors (at least Masters’s degree level or equivalent) trained to ensure adherence to the study protocol.

### Additional consent provisions for collection and use of participant data and biological specimens {26b}

Not applicable as informed consent is obtained for the RCT and the two sub-studies separately.

### Interventions

#### Explanation for the choice of comparators {6b}

Being the first RCT to test the effectiveness of this adjusted MB-PC program, we intend to test against enhanced standard clinical care, i.e., “treatment as usual,” which is directed against the MI itself and which is enriched by psychoeducation on parenting competences. The expected effect on parenting practices of such a workshop is very small which is why it is suitable as a control intervention in a first RCT. Treatment of the MI according to standard clinical care can have a positive effect on the parent-child relationship. However, a placebo group without psychiatric treatment (i.e., a waiting list) is ethically not acceptable for patients with a severe MI in need for acute treatment.

#### Intervention description {11a}

##### Mentalization-based parenting-counseling

Mentalization-based parenting-counseling (MB-PC) was developed with regard to mentalization-based therapy [40], borrows from the Lighthouse Parent Program against child maltreatment [41], and the mentalization-based primary prevention program [42]. MB-PC consists of a structured interview, video feedback, setting a focus, group therapy, psychoeducation, and social counseling. As the comparator intervention, MB-PC is also offered as an add-on to standard clinical care of the MI.

The 12-h manualized intervention program offered within 5 weeks of hospital treatment (covering standard clinical care) comprises five individual sessions assessing patients’ specific problems in parenting (2h), their experiences of being parented by their own parents (1h), and participation in a video interaction guidance with their child (2 × 1h). In a weekly 75-min therapeutic group, parents will learn to better understand and respond to the child’s mental needs and to cope with stigma related to MI. The group program consists of psychoeducation, reflection on diverse perspectives, and practicing through role-play as well as homework/exercises. Parents will be taught easy-to-learn metaphors about attachment and mentalization, e.g., parents are “lighthouses” that “illuminate” their children (mentalizing) and direct them to a “safe harbor” (secure attachment), sometimes through “rough seas” (e.g., MI). A focus will be on situations of distress when the “illuminating beam” is shut off and the risk of abuse is high. At the same time, parents will be supported in their existing parenting resources by highlighting their strengths. The 10 individual and group sessions will be supplemented by two sessions of social counseling (establishing family support, finding self-help groups and psychotherapists) taking place in weeks 4 and 5 of the intervention program. To keep close to routines in psychiatric hospitals, MB-PC will be provided by social workers and nurses specialized in psychiatry, who will be trained in MB-PC by experts. Closed groups will consist of 4–8 patients for reasons of practicability and facilitating group coherence. All participating therapists in MB-PC will be trained following a standardized training protocol. Supervision takes place every 2–3 weeks; additionally, therapists will meet for intervision once per week.

##### Control intervention (enhanced standard clinical care, SCC+)

Patients assigned to the control intervention take part in the standard clinical care directed at their MI at the Department of General Psychiatry at the University Hospital Heidelberg or at the Psychiatric University Hospital Charité at St. Hedwig Hospital. Additionally, they receive a 90-min workshop on parenting competences not based on mentalization-based therapy. The focus of this workshop is information on positive parenting, parental stress, and stress reduction. Additionally, the workshop entails two exercises on mindfulness. The control intervention is provided by different therapists (social workers and nurses) than the MB-PC.

#### Criteria for discontinuing or modifying allocated interventions {11b}

Before each individual session, patients will be asked about acute suicidality and significant deterioration of psychopathology attributed to the specific study intervention (see also the “Adverse event reporting and harms {22}” section) in which case a medical doctor of the participating hospitals will be informed to ensure treatment of acute suicidality and it will be decided whether participation in MB-PC will be stopped or the session will be postponed. Acute severe somatic illness that makes transfer to another medical department necessary is a further stopping reason.

#### Strategies to improve adherence to interventions {11c}

Therapist adherence to treatment manual will be ensured by regular supervision and will be controlled for by external coders on a random basis using the Revised Adherence Rating Scale [43] that is adapted to MB-PC, with all sessions being videotaped.

For patients, the intervention is part of their routine treatment schedule at the hospital.

#### Relevant concomitant care permitted or prohibited during the trial {11d}

Standard clinical care, i.e., “treatment as usual,” which is directed against the MI itself is provided and, thus, permitted during the trial. Standard clinical care subsumes pharmacotherapy, individual and group psychotherapy focusing on the given disorder, and supplementary creative and sports therapies.

#### Provisions for post-trial care {30}

Not applicable as the trial includes participants who are currently in treatment at a psychiatric hospital.

### Outcomes {12}

#### Primary outcome measure

**Fig. 1:**
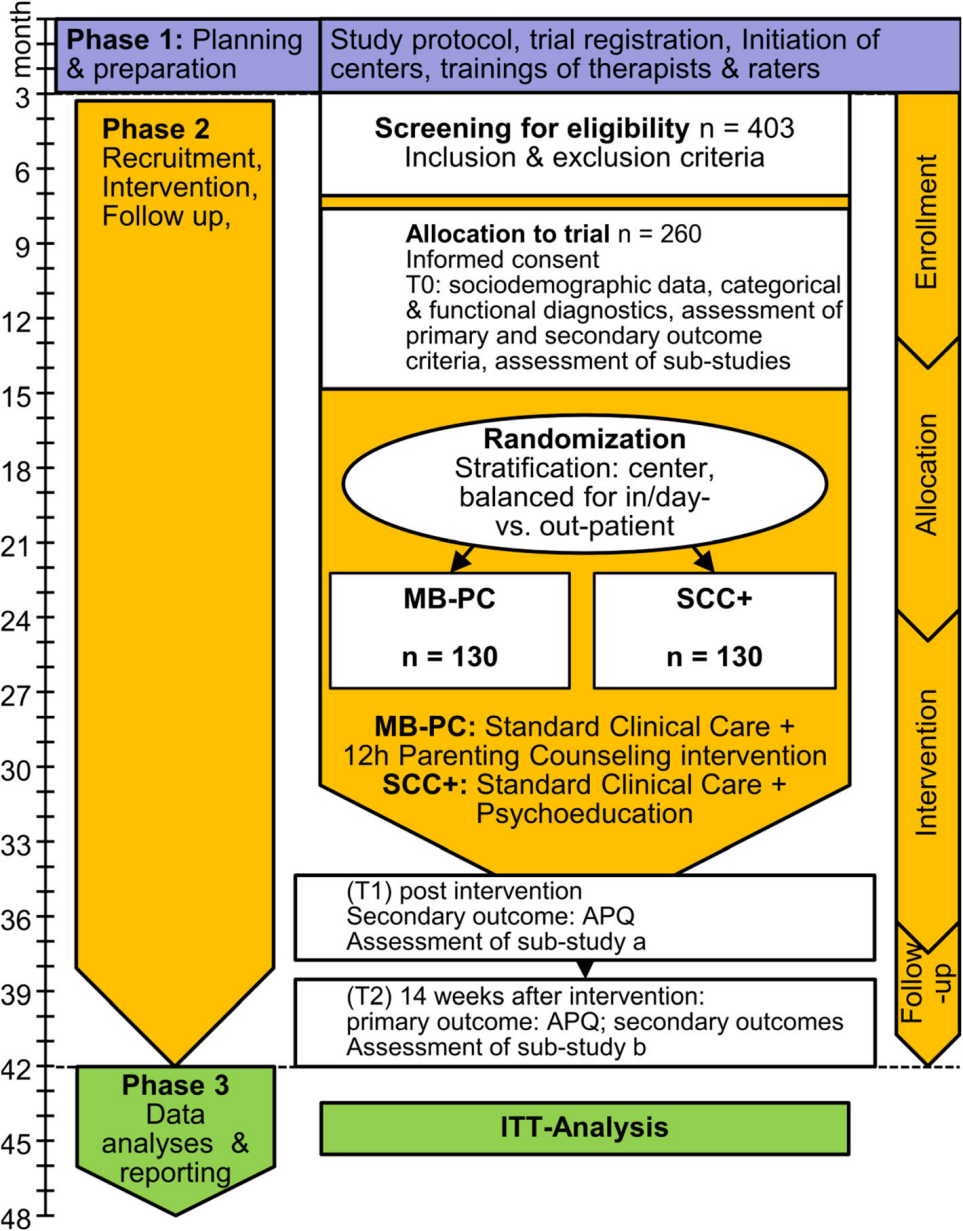
Trial flow

The primary endpoint of the RCT is parenting practices measured with the Alabama Parenting Questionnaire (validated German version) [44] at follow-up 14 weeks after the end of intervention (T2, see Fig. 1 and Table 1). A slightly modified version will be used for parents of children between 1.5 and 6 years in which items suitable only for older children are either rephrased (e.g., instead of “You ask your child about his/her day at school,” the modified item is “You ask your child about his/her day at kindergarten.”) or left out as missing values as they could not be rephrased accordingly (e.g., “Your child stays out in the evening past the time he/she is supposed to be home”). In all, of the total of 42 items, 15 items had to be rephrased and four items were omitted in the modified version. The version used for parents of children between 1.5 and 6 years in this study will be validated. The APQ will be administered at T0 (pre-intervention), T1 (post-intervention), and T2 (14 weeks after end of intervention) and is a reliable and valid 42 item self-report scale which measures parenting practices across five domains: parental involvement, positive parenting, poor monitoring/supervision, inconsistent discipline, and corporal punishment. It, thus, incorporates characteristics of parenting behaviors which are relevant for child development and conduct rather than emotional climate in the home and has been used as outcome measurement in several RCTs on parenting support programs (e.g., [45, 46]).

**Table 1:** Overview of the assessments at the different time points

Assessment	Study enrollment	Timepoint study period		
		T0 (pre-intervention)	T1 (post-intervention)	T2 (14 weeks after end of intervention)
Eligibility screen	x			
Informed consent patient and assent child	x			
Primary outcome measure Alabama Parenting Questionnaire (APQ)		x	x	x
Secondary outcome measures (self-report questionnaires on parental stress, parental abuse potential, parental mentalizing capacity, child psychopathology, and child well-being)		x		x
Other measures (diagnostic interviews and self-report questionnaires)		x		
Social cognition paradigms patients (behavioral and fMRI)		x	x	
Social cognition paradigms children 3years and older		x		x
Biobehavioral synchrony paradigm		x		x

#### Secondary outcome measures

Self-report questionnaires will be used to assess the secondary endpoints of the RCT, namely, parental stress (Parental Stress Index, PSI [47]), parental abuse potential (Child Abuse Potential Inventory, CAPI [48]), parental mentalizing capacity (Parental Reflective Functioning Questionnaire [49]), parenting and child rearing attitudes (Adult Adolescent Parenting Inventory, AAPI-2 [50]), parental emotion regulation (Difficulties in Emotion Regulation Scale, DERS [51]), and empathy (Interpersonal Reactivity Index, IRI [52]), all at T2. Additionally, child psychopathology (depending on the age of the child; 18–71 months: Child Behavior Checklist 1,5-5; starting from school enrollment: CBCL 6-18R [53]) and child well-being (KINDL [54, 55]) will be assessed at T2. Children will be asked to evaluate the parenting practices of mother and father using the APQ child version (from age 6), their well-being using the KINDL (from age 4) and their psychopathology using the Youth Self-Report Form (from age 11) [56]. Additionally, the parental APQ will be assessed as a secondary endpoint at end of intervention (T1, see Fig. 1 and Table 1), too.

Outcome measures sub-study a: On the behavioral level, outcome measures comprise behavioral indices for empathy, compassion, affect recognition, and ToM. On the neuronal level outcome measures include neuronal activities of the mentalizing and empathy networks (especially of the medial prefrontal cortex, anterior insula, ventral striatum, superior temporal sulcus bilateral temporo-parietal junction, and precuneus).

Outcome measures sub-study b: On the behavioral level, synchrony between parent and child according to the Coding Interactional Behavior-System (CIB [57]) is the outcome measure; physiological outcome measures include correlations between hormonal levels and heart rate variability of parent and child.

### Participant timeline {13}

At enrollment, patients will be screened for eligibility, and informed consent of the patient and, if applicable, assent of the child will be obtained. Patients can choose whether they take part in the intervention study only or also in one or two of the sub-studies. The intervention starts when a group of 4–8 patients is available which are randomized together to MB-PC or SCC+. Data assessment will take place before the beginning of the intervention (T0), at the end of the intervention (T1) and 14 weeks after end of intervention (T2).

Data assessment of sub-study a takes place at T0 and T1, of sub-study b at T0 and T2. See Table 1 for an overview of the assessments at the different time points.

### Sample size {14}

A recent meta-analysis of the effects of parenting programs [15] on child maltreatment prevention showed moderate effect sizes (total random effect size of *d*=0.3 in 37 studies), which vary by intervention program, target population, and outcome measure. Moderator analysis showed that programs applied in selected or indicated samples had stronger effects than those in universal samples. Effect sizes for the APQ subscales reported in the literature vary between small to moderate effect sizes and are reported for different samples and therapeutic interventions: In a study including a risk sample of formerly incarcerated mothers effect sizes for the subscales vary between *d*=0.09 and *d*=0.32 [45], in another study including parents at risk for physical abuse between *d*=0.32 and *d*=0.65 [46]. Other studies assessing the APQ included parents of children with behavioral problems [58] or attention deficit hyperactivity disorder [59] and report effect sizes for the subscales between *d*=0.03 and *d*=0.62 with the majority of the subscales showing effect sizes between *d*=0.41 and *d*=0.49. Based on these results, we expect in this selected sample and for the APQ, an effect size of 0.35 to 0.45. A sample size calculation based on a two-sided *t*-test (significance level *α*=5) with a power of 80% for a superiority test with an alternative of an expected effect size of *d*=0.4 (difference in change in the primary outcome measure APQ from T0 to T2 between MB-PC and SCC+ arms) leads to a required sample size of 100 patients per group (using G*Power 3.1.9.2); for a true effect size of *d*=0.45 or *d*=0.35, this sample size would lead to 89% or 69% power. Note that the actual primary analysis will be multivariate for the five APQ-subscales and adjusted for covariates (see below), which is expected to provide even higher power. We expect low intra-cluster correlation (ICC; within intervention groups as used in clustered randomization or by therapists delivering the intervention), as in MB-PC 7 of 12 h are individual sessions and only 5 h are in a group-setting and the control intervention workshop does not contain any specific group exercises. However, to account for a potential small ICC of 0.02 to 0.05 [60] together with the expected cluster size (size of therapy group) of N=5, this yields a variance inflation factor (VIF) of 1.08 to 1.2. We decided to use a VIF of 1.1, thus increasing sample size by 10%. Assuming additionally up to 15% of randomized patients without any data post-baseline leads to a required sample size of 260 randomized patients.

Regarding the two sub-studies, based on power estimates and sample size calculation for mediation analyses [61], we may expect sufficient power (>80%) with a sample size of 177, respectively 120 patients, if there are small to medium direct and mediator effect sizes among the different predictor, outcome, and mediator variables.

### Recruitment {15}

Following the design of a pragmatic RCT in “real-world” clinical settings, all mothers and fathers of 1.5–15-year-old children with regular contact to their children, consecutively admitted to psychiatric hospital care at the Department of General Psychiatry at the University Hospital Heidelberg and the Psychiatric University Hospital Charité at St. Hedwig Hospital in Berlin, will be asked to participate in the trial. Furthermore, patients from the psychiatric institutional outpatient departments (PIA) offering high-frequent multi-professional treatment for patients with severe mental disorders are included. The populations in the two psychiatric hospitals differ in socioeconomic status (SES), with the University Hospital Heidelberg being located in a medium-sized high-SES town and the Psychiatric University Hospital Charité at St. Hedwig Hospital in a metropolitan region with a rather low SES, socially troubled areas and high rates of immigrants. The sample will hence be representative for urban German psychiatric care, making the outcomes essential for future clinical decisions in broad segments of the population. At each of these two study sites 130 patients will be recruited to take part in the RCT. Patients that take part in the RCT will be asked to participate in the sub-studies. The study on social cognition will recruit 104 patients at each site, the study on biobehavioral synchrony will recruit 73 patients with children aged 3–15 at each site. The sub-studies will include 30 healthy controls at each study site, too. The trial flow is shown in Fig. 1.

## Assignment of interventions: allocation

### Sequence generation {16a}

Patients are randomised in clusters of 4–8 patients who will have all their group sessions together. The allocation sequence of intervention groups to the two arms will be based on computer-generated random numbers. Randomization will be first stratified by center (Heidelberg and Berlin, each group consists only of patients from one centre) and within center balanced for treatment setting (in- or day-patient vs. outpatient, each group can consist of patients of all of these settings), using a dynamic minimization algorithm [62], which is adapted for cluster-randomization [63]. In the original version of this algorithm, the next unit to be randomised is assigned in a deterministic way to one arm (based on current imbalance); however, to make the randomization less predictable and ensure concealment of allocation, this is amended by a biased-coin design [64].

### Concealment mechanism {16b}

Concealed randomization after definite inclusion of a complete group of 4–8 patients will be performed by IMIS Kiel, via e-mail. The IMIS Kiel is not involved in recruitment, assessments or therapy of the patients.

### Implementation {16c}

The allocation sequence will be generated by the IMIS Kiel. The IMIS Kiel will also assign participant groups to interventions. At each study center study personnel not involved in recruitment, data assessment or therapy of the patients will inform the patients about the dates of the group sessions (either for MB-PC and for SCC+).

## Assignment of interventions: Blinding

### Who will be blinded {17a}

Patients and therapists cannot be blinded regarding treatment allocation in a psychological intervention. The primary outcome measure will be obtained from self-report (thus cannot be assessed in a blinded way). Since patients will be ensured that their ratings will not become known to therapists, we expect no relevantly biased assessment of results to occur. All personnel involved in recruitment and data assessment including experimental sessions will be blinded regarding treatment allocation of the patients.

### Procedure for unblinding if needed {17b}

Not applicable as patients and therapists cannot be blinded regarding treatment allocation (see the “Who will be blinded {17a}” section).

## Data collection and management

### Plans for assessment and collection of outcomes {18a}

In addition to the primary and secondary outcome measures (see above), the following data will be assessed: Sociodemographics (i.e., gender, age of parent and child, socioeconomic status, etc.); interviews administered by experienced diagnosticians (master’s degree in psychology or equivalent) will be used to assess Axis I disorders (Diagnostic Short Interview for Mental Disorders; Mini DIPS) [65], Borderline Personality Disorders (International Personality Disorder Examination, IPDE) [66] and levels of personality functioning (brief form of semi-structured Interview for Personality Functioning; StiP) [67]. Diagnosticians will also evaluate the global functioning according to the Global Assessment of Functioning, GAF [68]. Furthermore, self-report questionnaires will be used to assess symptom severity (Brief-Symptom-Inventory, BSI-53) [69], depressiveness (BDI) [70], early life maltreatment (Childhood Experience of Care and Abuse Questionnaire, CECA-Q [71]; Childhood Trauma Questionnaire, CTQ) [72], and attachment (Relationship Structures Questionnaire, ECR-RS, [73]. The mini-q [74] will be used to assess cognitive capacity. All questionnaires and semi-structured interviews are widely used in psychiatric research and have good reliability and validity.

Study a comprises four behavioral tasks and three fMRI tasks on five different aspects of social cognition: empathy, compassion, affect recognition, and affective and cognitive ToM. We use well-established tasks slightly changed in design to fit the purpose of the present study (i.e., showing pictures of children instead of adults). Parents will be asked to perform four computer-based tasks: first, a photo-task to study empathy and compassion, in which subjects view photos of affective child scenarios; second, a morphing task to study affect recognition [75]; third, an empathy and compassion task (adopted from [76]) in which subjects view affective child scenarios; and finally, a video task (adopted from [77]) assessing compassion and cognitive ToM, in which subjects will be presented with short videos of adults describing situations of emotionally neutral or negative content. In the MR scanner, subjects will be asked to perform three tasks: first, a ToM task, in which subjects view drawings of children in different affective situations; second, a pain empathy task (adopted from [78]), in which photos of children in physical painful or non-painful situations will be shown; and, lastly, a face task (adopted from [79]) in which specific statements followed by child faces in different emotional expressions will be shown (happy, sad, afraid, angry) and subjects will have to judge whether the depicted statement matches the picture of the child.

Study b uses a standardized experimental design to assess biobehavioral dyadic synchrony:

Biobehavioral synchrony will be measured within a parent-child interaction paradigm at T0 and T2. This paradigm includes a positive dyadic interaction (i.e., planning a positive joint activity) as well as a stressful dyadic interaction (i.e., solving unsolvable puzzles under time pressure) under controlled laboratory conditions. On the behavioral level, synchrony between parent and child will be measured using the Coding Interactional Behavior-System (CIB; Feldman, 1998). On the biological level, parent-child synchrony of both autonomous nervous system (ANS) activity (i.e., heart rate and heart rate variability) and hormonal release (i.e., cortisol and oxytocin) will be assessed. Study b further administers behavioral tasks on social cognition (empathy, compassion, affect recognition, and ToM) adapted to the age of the child. Children from 3 to 6 years of age will first be presented selected tasks from the Strange Stories task battery [80] and the Extended Theory-of-Mind Scale [81] and the complete Test of Emotion Comprehension [82] to assess ToM. Second, the “Affect Recognition” scale from the NePsy (“A Developmental NEuroPSYchological Assessment” [83]) will be administered. To assess empathy in preschool children, parents will be asked to complete the Empathy Questionnaire (EmQue [84]). Children from 7 to 15 years of age will first perform an almost identical version of the photo-task described above (parental assessment) to study empathy and compassion. Second, to assess ToM-selected tasks from the Strange Stories task battery [80] and a range of advanced ToM tasks will be administered [85]. Third, an almost identical version of the morphing task as described above (parental assessment) to study affect recognition [75] with age adapted instructions will be presented.

### Plans to promote participant retention and complete follow-up {18b}

All participants will be asked to participate in the post-intervention (T1) and follow-up assessments (at T2), even if they drop out of treatment, in order to minimize the number of those lost to follow-up. To keep missing data low, we will reimburse patients for the assessment of primary and secondary outcomes and will offer to visit patients at home at T2.

### Data management {19}

Data management is administered via a central data bank (Redcap). Self-report questionnaires are completed online via Lime-Survey, also interviews and measurement reports have been digitalized and made available online, all information for evaluations was compiled, and evaluation routines for all questionnaires were written prior to data collection. All details of data management procedures can be found in a separate study plan available to all study personnel.

### Confidentiality {27}

All data are subject to medical confidentiality and will be handled in accordance with the European Union General Data Protection Regulation (DSGVO) and with the German legal regulations regarding data protection and security (Landesdatenschutzgesetz Baden-Wuerttemberg, Bundesdatenschutzgesetz). All data will be pseudonymised and transferred to a server of the company netcup (Nuremberg, Germany), the transfer will be encrypted. Access to the data will be strictly limited to authorized persons and will be password-protected. Data will be stored for 10 years.

### Plans for collection, laboratory evaluation, and storage of biological specimens for genetic or molecular analysis in this trial/future use {33}

Not applicable as no biological specimens were collected as part of this trial.

## Statistical methods

### Statistical methods for primary and secondary outcomes {20a}

Superiority of the MB-PC intervention regarding APQ scores at T2 compared to SCC+ will be tested at a two-sided significance level of 5%. In the primary analysis, we will use data of both the T1 and T2 assessments (to estimate treatment contrast at T2) in a linear mixed-effects model, with all five APQ subscores as dependent variables in a multivariate model to test for global differences between arms, followed by appropriate post hoc tests for the subscores individually [86]. The analysis will include the covariates used for randomization: center, and treatment setting, and as additional covariates the gender of parent and child, the age of child, the baseline value (T0) of the primary outcome (all 5 APQ subscores), and the primary ICD-11 diagnosis at T0. Analyses of secondary outcomes will be conducted analogously. Safety analyses: Frequencies of AEs and SAEs in all randomized participants will be tabulated, separately for intervention and control group and by presumed relation to intervention and severity.

Statistical analysis will use the software R (http://www.R-project.org). All details will be specified in the statistical analysis plan prior to any data analysis.

### Interim analyses {21b}

There will be no interim analysis.

### Methods for additional analyses (e.g., subgroup analyses) {20b}

We will perform subgroup analyses in patients with and without ELM for primary and secondary outcomes.

To test the hypothesis that MB-PC-related behavioral and neural changes in social cognition as well as changes in biobehavioral synchrony will mediate the effects of the MB-PC intervention, we will test the indirect effect of MB-PC (proposed predictor) on outcome scores via parental social cognition and via biobehavioral synchrony (proposed mediators), respectively in a multiple mediator analysis using ordinary least squares regressions with bootstrapping.

To test the hypothesis that ELM will moderate the effects of MB-PC on social cognition and biobehavioral synchrony, we will calculate generalized linear (mixed) models with history of ELM (yes/no) and intervention group (MB-PC versus SCC+) as between-subject factors. A positive interaction between the factors intervention and ELM will indicate that MB-PC exerts particularly high effects in parents affected by ELM.

### Methods in analysis to handle protocol non-adherence and any statistical methods to handle missing data {20c}

The primary analysis will be based on the intention-to-treat (ITT) principle, aiming to include all randomized patients in the arm they were originally randomised to and irrespective of the amount of treatment actually received. Sensitivity analyses will be performed to investigate the potential impact of missing data, in particular by using pre-specified conservative multiple imputation strategy and a complete case analysis. A per-protocol (PP) analysis will also be performed as sensitivity analysis. Deviations from the protocol that lead to exclusion from the PP set will be detailed in the statistical analysis plan, which will be written prior to any data analysis.

### Plans to give access to the full protocol, participant-level data, and statistical code {31c}

We will offer access to the full protocol, participant-level dataset, and statistical code on demand.

## Oversight and monitoring

### Composition of the coordinating center and trial steering committee {5d}

The Coordination Center for Clinical Trials (KKS) Heidelberg will monitor the study according to its SOPs and adapted to ICH-GCP guideline E6 (R2) to ensure adherence to ethical aspects, patient’s rights, and quality of data documentation.

### Composition of the data monitoring committee, its role, and reporting structure {21a}

An independent Data Safety and Monitoring Board (DSMB) will monitor and supervise the progress of the trial as well as the adherence to protocol. The board will supervise safety data and will be informed about severe adverse advents. In addition, the Scientific Advisory Board of the consortium will be asked for advice in the case of recruitment fails at any site and they should be prepared to provide the funding agency with information. Prof. Dr. Susanne Walitza, Child and Adolescent Psychiatry University of Zuerich; Prof. Dr. André Scherag, Institute of Medical Statistics, Computer and Data Sciences, University Hospital Jena; and Prof. Dr. Gerhard Suess, University of Applied Sciences, Hamburg, are part of the DSMB.

### Adverse event reporting and harms {22}

Severe adverse events (SAE) comprise child endangerment during the intervention period, suicide or suicide attempt with necessity of treatment at a closed ward or a somatic unit during the intervention period and suicide or suicidality with necessity of treatment at a closed ward after dismissal from hospital care until T2. In case of an SAE, a medical doctor of the participating hospitals will be informed to ensure treatment of acute suicidality. Furthermore, the principal investigator will be informed and will subsequently inform the DSMB.

### Frequency and plans for auditing trial conduct {23}

During the course of the study, two monitoring visits per trial site will take place in order to verify patient safety and data integrity employing a risk-based approach. Monitoring will be performed by the Coordination Centre for Clinical Trials (KKS) at Heidelberg University Hospital, the monitor is independent of the investigators. The trial might be audited by the sponsor quality management unit (SpoQS) of the Heidelberg University Hospital; auditors are independent of the investigators.

### Plans for communicating important protocol amendments to relevant parties (e.g., trial participants, ethical committees) {25}

Protocol modifications will be submitted to the Ethics Committee of the Medical Faculty at the University of Heidelberg. The managing site at the Department of General Psychiatry at the University Hospital Heidelberg will then communicate the changes to the German Clinical Trials Register. Each study site is responsible in training their study personnel in protocol modifications.

## Dissemination plans {31a}

Results will be published in research publications and conference contributions. In order to disseminate the results beyond the scientific community, the UBICA-II consortium will not only inform study participants via a newsletter about the findings from the study, but the consortium also cooperates with several self-help groups and initiatives throughout Germany (Wildwasser Esslingen e. V., Shadow and Light – Initiative for Peripartum Mental Disorders, Self-Help group Zwickmuehle, Federal Centre for Early Prevention) who have expressed their support of the study. Close cooperation with a local self-help group (Self-Help group Zwickmuehle) allows to integrate peer support in addition to experts’ support in the MB-PC program. We will inform health care providers about the results of this project via the German Association of Psychiatrists (DGPPN) and the German Society of Child Psychiatry and Psychotherapy (DGKJP) as the scientific societies, and the Bundesdirektorenkonferenz and Bundesarbeitsgemeinschaft leitender Klinikaerzte (BDK, BAG) and the Association of Psychiatrists at General Hospitals (ACKPA) as the legal advocacies of psychiatric and child psychiatric hospitals. Based on the results, we plan to disseminate our preventive approach by offering in-house training for relevant institutions of the German welfare (e.g., Early Prevention System) and health system (hospitals) to convey team-based expertise in addition to expert training.

## Discussion

This is the first study to implement MB-PC as an add-on to routine psychiatric hospital care in Germany. It includes MI parents with a focus on those with a history of ELM and thus reaches families with the highest risk of child neglect and abuse. If evidence of effectiveness is found, MB-PC can be directly implemented into standard hospital treatment. The implementation is favored by low costs and takes into account the typical staffing in German psychiatric hospitals. The individual patient participating in MB-PC is expected to immediately benefit from the trial. Besides establishing and evaluating new interventions, it is important to understand the behavioral and neural mechanisms of change induced by this MB intervention and the effect modification by ELM. The present study therefore not only tests the effectiveness of MB-PC but also investigates possible mediators and moderators in sub-studies. Based on the knowledge on such mechanisms of change it will be possible to further improve the intervention and to tailor it to the needs of the affected parents.

The bicenter study has many advantages (such as recruiting participants with a broad range of mental disorders in different socioeconomic backgrounds in Heidelberg and Berlin) but also comes with special demands regarding standardized recruitment and implementation procedures. For this reason, a reliable and close cooperation between the study centers is highly important. To ensure standardization of procedures, staff from both study sites were instructed in joint trainings prior to the start of recruitment to ensure adherence to the treatment manual and the study protocol. Furthermore, a joint databank and detailed protocols for all study parts shall ensure the standardization of procedures at both study sites.

Despite MB-PC and the workshop in SCC+ concentrating on different aspects of parenting (e.g., SCC+ only focuses on positive interactions while MB-PC also takes into account difficult interactions between parent and child) and using specific outcome measures, we cannot completely rule out a possible dose effect. This is due to the present study being the first study to test effectiveness of MB-PC as an add-on to routine psychiatric hospital care and therefore testing it against SCC+. Future studies should compare MB-PC to other, more extensive parenting programs (if available by that point).

### Future impact

During psychiatric treatment, low-threshold access to treatments strengthening parenting abilities is given for severely ill MI parents. Interventions in this population are urgently needed in order to prevent abuse or neglect in children of parents at high risk of child maltreatment.

If MB-PC is shown to be effective, it can be directly and cost-effectively implemented into standard psychiatric hospital care. The present study therefore has the potential to improve the mental health care system, to prevent child maltreatment and to offer a possibility to break the intergenerational cycle of abuse.

## Trial status

Protocol version number and date: Version 1.6, April 26, 2021.

Start of recruitment: 08/2019; expected end of recruitment: 08/2022.

## Data Availability

All investigators will have access to the final trial dataset.

## Abbreviations

MI: Mental illness
ELM: Early life maltreatment
MB-PC: Mentalization-based parenting-counseling
SCC: Standard clinical care
RCT: Randomized controlled trial
ToM: Theory of mind
CIB: Coding interactional behavior-system
fMRI: Functional magnetic resonance imaging
ANS: Autonomous nervous system
AE: Adverse event
SAE: Severe adverse event
ITT: Intention-to-treat
DSMB: Data safety and monitoring board
